# Nicotine Self-Administration Induces Plastic Changes to Nicotinic Receptors in Medial Habenula

**DOI:** 10.1523/ENEURO.0197-20.2020

**Published:** 2020-08-03

**Authors:** Xiao-Tao Jin, Brenton R. Tucker, Ryan M. Drenan

**Affiliations:** Department of Physiology and Pharmacology, Wake Forest University School of Medicine, Winston-Salem, NC 27157

**Keywords:** acetylcholine, addiction, habenula, nicotine, relapse, tobacco

## Abstract

Chronic nicotine upregulates nicotinic acetylcholine receptors (nAChRs) throughout the brain, and reducing their activity may promote somatic and affective states that lead to nicotine seeking. nAChRs are functionally upregulated in animal models using passive nicotine administration, but whether/how it occurs in response to volitional nicotine intake is unknown. The distinction is critical, as drug self-administration (SA) can induce neurotransmission and cellular excitability changes that passive drug administration does not. In this study, we probed the question of whether medial habenula (MHb) nAChRs are functionally augmented by nicotine SA. Male rats were implanted with an indwelling jugular catheter and trained to nose poke for nicotine infusions. A saline SA group controlled for non-specific responding and nicotine-associated visual cues. Using patch-clamp whole-cell recordings and local application of acetylcholine, we observed robust functional enhancement of nAChRs in MHb neurons from rats with a history of nicotine SA. To determine whether upregulated receptors are generally enhanced or directed to specific cellular compartments, we imaged neurons during recordings using two-photon laser scanning microscopy (2PLSM). nAChR activity at the cell soma and on proximal and distal dendrites was examined by local nicotine uncaging using a photoactivatable nicotine (PA-Nic) probe and focal laser flash photolysis. Results from this experiment revealed strong nAChR enhancement at all examined cellular locations. Our study demonstrates nAChR functional enhancement by nicotine SA, confirming that volitional nicotine intake sensitizes cholinergic systems in the brain. This may be a critical plasticity change supporting nicotine addiction.

## Significance Statement

This study demonstrates that stable, volitional nicotine intake is sufficient in dosage and duration to robustly enhance nicotinic acetylcholine receptor (nAChR) functional activity in medial habenula (MHb), a brain area involved in addiction, anxiety, and fear memory. Human tobacco use, via cigarettes or electronic nicotine delivery systems (i.e., e-cigarettes), is also likely to induce such changes to nAChRs. Minimal nicotine exposure is required to induce MHb nAChR upregulation, suggesting that it occurs early in the addiction process and that the behavioral response to repeated nicotine will include a contribution from these upregulated receptors. Available drugs and current regulatory policy are not optimal for fostering tobacco cessation, highlighting the importance of identifying mechanisms that enable continued tobacco use.

## Introduction

Tobacco product usage leads to more cases of preventable death than any other human activity (CDC, 2018). In the United States, the economic costs of smoking-related illness exceed $300 billion annually (DHHS, 2014; Xu et al., 2015). This amounts to ∼40% of the United States’ annual defense budget and >700% of the annual budget of the National Institutes of Health. Electronic nicotine delivery system (i.e., vaping, JUULing, etc.) use is on the rise among American youth, which could lead to a new generation of cigarette smokers (Young-Wolff et al., 2018). Current smoking cessation pharmacotherapies, such as bupropion and varenicline, have not been adequate to enable tobacco users to quit and remain tobacco-free. Addiction to nicotine, the primary reinforcing agent in tobacco, is necessary for sustained tobacco usage. Understanding the neurobiological mechanisms of nicotine’s action in the brain, using valid preclinical animal models of nicotine consumption, could promote new smoking cessation therapeutic development as well as inform public policy.

It is well established that repeated or prolonged nicotine exposure causes nicotinic acetylcholine receptors (nAChRs), the pharmacological target of nicotine, to increase in number on the cell surface and within cells (Marks et al., 1983; Schwartz and Kellar, 1983). This process, also known as nAChR upregulation, occurs in tissue culture cells (Peng et al., 1994), invertebrates (Feng et al., 2006), rodents (Marks et al., 1983; Schwartz and Kellar, 1983), non-human primates (McCallum et al., 2006), and, most importantly, in human smokers (Mukhin et al., 2008). nAChR upregulation is an important plastic change supporting addiction to nicotine. In particular, upregulation is likely involved in sensitization (Tapper et al., 2004), enhanced nicotine-mediated activation of the dopamine system (Rice and Cragg, 2004; Zhang and Sulzer, 2004; Nashmi et al., 2007), and severity of depression episodes during withdrawal (Mineur et al., 2013). Upregulation is also paradoxical (Wonnacott, 1990), as other drug receptors are typically reduced in numbers when chronically exposed to agonist (Creese and Sibley, 1981). In some instances, nAChR upregulation has been suggested to be a compensatory response to nicotine-mediated desensitization (Schwartz and Kellar, 1983; Lapchak et al., 1989), implying that upregulated receptors are not functional.

Although nAChR upregulation is understood to be a feature of nicotine exposure, a number of knowledge gaps exist. For example, a majority of animal studies of nicotine-induced nAChR upregulation have employed passive, non-contingent exposure paradigms such as experimenter-administered injections, osmotic minipumps, or inclusion of nicotine in the drinking water. Although such methods are technically approachable and expend fewer resources, the pharmacokinetics of nicotine in these models does not replicate the exposure parameters in models of volitional nicotine intake such as intravenous self-administration (SA; Matta et al., 2007). This is an important distinction, as non-contingent versus contingent nicotine exposure can lead to different plasticity changes (Caillé et al., 2009; Metaxas et al., 2010). Studies that have documented nAChR upregulation in nicotine SA models (Donny et al., 2000; Le Foll et al., 2016) have relied on methods (e.g., radioligand binding, quantitative immunohistochemistry, etc.) that are unable to examine nAChR functional activity. Such methods do not provide the spatial resolution required to understand the potential single-cell specificity of nAChR plasticity. They have also failed to detect upregulation altogether. For example, radioligand binding (Huang and Winzer-Serhan, 2006) and immunohistochemical (Nashmi et al., 2007) approaches failed to detect nAChR upregulation in medial habenula (MHb), a small brain region critical for nicotine dependence (Fowler et al., 2011; Zhao-Shea et al., 2013). Subsequently, we used electrophysiological measurements to demonstrate robust MHb nAChR functional upregulation in two different non-contingent exposure paradigms (Shih et al., 2015; Banala et al., 2018; Arvin et al., 2019a). Blockade of ongoing MHb nAChR activity is sufficient to trigger withdrawal behavior in mice exposed chronically to nicotine but not saline (Salas et al., 2009), which strongly implicates MHb nAChR upregulation.

In the present study, we addressed these gaps by examining the impact of nicotine SA on nAChR functional activity in the MHb. Adult male rats that acquired stable nicotine intravenous SA were used to prepare *ex vivo* brain slices for patch-clamp recordings of acetylcholine-evoked nAChR responses. Additionally, two-photon laser scanning microscopy (2PLSM) and nicotine uncaging was used to probe nAChR activity at discrete locations on the cell membrane.

## Materials and Methods

### Materials

Picrotoxin (PTX), atropine sulfate (atropine), acetylcholine chloride, and 4-aminopyridine (4AP) were obtained from Sigma. 6-Cyano-7-nitroquinoxaline-2,3-dione (CNQX) and D-(-)−2-amino-5-phosphonopentanoic acid (D-AP5) were obtained from Tocris. QX314 chloride (QX314) was from EMD-Millipore. Tetrodotoxin citrate (TTX) was obtained from Abcam. Nicotine hydrogen tartrate salt was obtained from Glentham Life Sciences. Injectable heparin sodium was from Patterson Veterinary Supply.

### Rats

All experimental protocols involving rats were reviewed and approved by the Wake Forest University institutional animal care and use committee. Procedures also followed the guidelines for the care and use of animals provided by the National Institutes of Health Office of Laboratory Animal Welfare. All efforts were made to minimize animal distress and suffering during experimental procedures, including during the use of anesthesia. Male Sprague Dawley rats (Envigo; total *n* = 31) were ∼300 g (approximately eight weeks old) when they arrived at our facility. Only males were used in this study, for the following reasons: (1) only one housing room was readily available, and (2) no females could be housed in the same room with males without negatively impacting the behavioral data. Rats were housed at 22°C on a reverse 12/12 h light/dark cycle (4 P.M. lights on, 4 A.M. lights off).

### Apparatus

Rats were trained in Med Associates operant chambers (interior dimensions, in inches: 11.9 × 9.4 × 11.3) located within sound-attenuating cabinets. The SA system was housed in a dedicated room within the same laboratory suite as the rat’s housing room. A PC computer was used to control the SA system via Med PC IV software. Each chamber had clear Plexiglas walls, a stainless-steel grid floor, and was equipped on the right-side wall with two nose pokes (2.4 inches from grid floor to nose poke center) which flanked a pellet receptacle coupled to a pellet dispenser. A white stimulus light was located above each nose poke, and a house light was located at the top of the chamber on the left-side wall. During food and drug SA sessions, nose pokes on the active nose poke activated either the pellet dispenser or an infusion pump, respectively. Nose pokes on the inactive nose poke had no consequence. For intravenous drug infusions, each rat’s catheter was connected to a liquid swivel via polyethylene tubing that was protected by a metal spring. The liquid swivel was connected to a 10-ml syringe loaded onto the syringe pump.

### Operant food training

Approximately one week after arrival, rats were food restricted for several days to enhance their motivation to participate in operant training. This involved feeding rats 20-g standard chow once per day rather than *ad libitum* feeding. Water was available *ad libitum* except during operant behavioral sessions, when no water was available. Food training sessions were 1 h in duration, and rats were trained to nose poke for food pellets (45 mg; Bio-Serv Dustless Precision Pellets, catalog #F0021) on the same nose poke that would subsequently be paired with drug infusions in nicotine IVSA sessions. A fixed ratio 1 (FR1; no timeout) schedule was used for food training, where no visual cues (stimulus light, house light) were illuminated during the session and rats could earn a maximum of 75 food pellets during the 1-h session. Once each rat successfully earned at least 50 pellets with at least a 2:1 preference for the active nose poke over the inactive nose poke, no further food training was conducted. Rats met this criterion within one to three sessions.

### Indwelling jugular catheter surgery

After acquisition of food operant responding, rats were anesthetized with isoflurane (5% induction, 2–3% maintenance) and implanted with indwelling jugular catheters. Lidocaine (1–2 mg/kg; intradermal and “splash block”) was used during incision closure to improve recovery, and ketoprofen (2–3 mg/kg) or meloxicam (2 mg/kg) was administered postoperatively to relieve pain and reduce inflammation. Rats were singly housed following surgery and throughout all SA procedures. Rats were allowed 7 d for recovery from surgery, and catheters were flushed several times during this recovery period with heparin sodium dissolved in sterile saline.

### Intravenous drug SA

After recovery from catheter surgery, rats were allowed to self-administer saline or nicotine (0.03 mg/kg free base/infusion) in a volume of 0.035 ml over 2 s during 2-h SA sessions, Monday through Friday (no SA sessions occurred on weekends). SA parameters were modeled primarily on pioneering studies by Corrigall (Corrigall and Coen, 1989) and Donny (Donny et al., 1995). (-)-Nicotine hydrogen tartrate salt (Glentham Life Sciences) was dissolved in sterile saline, and the pH was adjusted to 7.4. For saline SA, the same sterile saline vehicle was pH-adjusted to 7.4 and used as the intravenous drug solution. Infusions, delivered by an infusion pump, were triggered by one nose poke response (FR1) on the active nose poke. Infusions (2 s in duration) were simultaneously paired with illumination of the stimulus light over the active nose poke for 3 s. An active nose poke response that resulted in an infusion extinguished the house light for a 20-s timeout period, during which responding was recorded but had no consequence. Responses on the inactive nose poke were recorded but had no scheduled consequence. At the end of the session, the house light was extinguished and responding had no consequences. Rats were removed from the training chambers as soon as possible after the end of the 2-h session. Rats were allowed to self-administer saline or nicotine for up to 15 sessions. After the first five SA sessions, which were needed for the novelty effects of operant behavior sessions to wane, responding for nicotine was evaluated on a daily basis. Rats were removed from the study if (1) a dramatic (>75%) drop in responding on the active nose poke occurred and this reduced responding was sustained for two or more days, or (2) the ratio of active to inactive responses was less than 2 for 3 consecutive days. Of *n* = 16 rats in the nicotine SA group that were catheterized, one failed to acquire SA (based on the above criteria), and his data are not included in the study. One injured his leg on day 7, so data from this rat up to/including day 6 are included. One lost patency on day 11, so the data from this rat are included up to/including day 10. Of *n* = 12 rats in the saline SA group that were catheterized, none lost patency and all data from these rats are included in the study.

A yoked saline control group (*n* = 3) was also prepared as an additional control for electrophysiology studies. For this group, rats were food restricted, food trained, and implanted with jugular catheters as described above. During 2-h yoked saline sessions, nose poking had no consequence and saline was non-contingently delivered 16 times through the catheter according to a predetermined time schedule that was derived from a previous rat’s nicotine SA session. This approach was preferable to a true/classical “yoked” delivery as it enabled somewhat greater flexibility. This group allowed us to determine whether there were any appreciable effects of the nicotine-associated visual cues on our measured physiological endpoints.

### Brain slice preparation and recording solutions

Rats were anesthetized with isoflurane before trans-cardiac perfusion with oxygenated (95% O_2_/5% CO_2_), 4°C *N*-methyl-D-glucamine (NMDG)-based recovery solution that contains the following: 93 mm NMDG, 2.5 mm KCl, 1.2 mm NaH_2_PO_4_, 30 mm NaHCO_3_, 20 mm HEPES, 25 mm glucose, 5 mm sodium ascorbate, 2 mm thiourea, 3 mm sodium pyruvate, 10 mm MgSO_4_·7H_2_O, and 0.5 mm CaCl_2_·2H_2_O; 300–310 mOsm; pH 7.3–7.4. Brains were immediately dissected after the perfusion and held in oxygenated, 4°C recovery solution for 1 min before cutting a brain block containing the MHb and sectioning the brain with a vibratome (VT1200S; Leica). Coronal slices (250 μm) were sectioned through the MHb and transferred to oxygenated, 33°C recovery solution for 12 min. Slices were then kept in holding solution containing the following: 92 mm NaCl, 2.5 mm KCl, 1.2 mm NaH_2_PO_4_, 30 mm NaHCO_3_, 20 mm HEPES, 25 mm glucose, 5 mm sodium ascorbate, 2 mm thiourea, 3 mm sodium pyruvate, 2 mm MgSO_4_·7H_2_O, and 2 mm CaCl_2_·2H_2_O; 300–310 mOsm; pH 7.3–7.4 for 60 min or more before recordings. Brain slices were transferred to a recording chamber (1-ml volume), being continuously superfused at a rate of 1.5–2.0 ml/min with oxygenated 32°C recording solution. For our recording chamber and solution flow rate, we estimate that complete solution exchange occurs in 5–8 min. The recording solution contained the following: 124 mm NaCl, 2.5 mm KCl, 1.2 mm NaH_2_PO_4_, 24 mm NaHCO_3_, 12.5 mm glucose, 2 mm MgSO_4_·7H_2_O, 2 mm CaCl_2_·2H_2_O, 0.01 mm CNQX, 0.03 mm D-AP5, and 0.1 mm PTX; 300–310 mOsm; pH 7.3–7.4). For puffer experiments only, the recording solution was supplemented with 1 μm atropine. Patch pipettes were pulled from borosilicate glass capillary tubes (1B150F-4; World Precision Instruments) using a programmable microelectrode puller (P-97; Sutter Instrument). Tip resistance ranged from 7.0 to 10.0 MΩ when filled with internal solution. A potassium gluconate-based internal solution was used for recordings: 135 mm potassium gluconate, 5 mm EGTA, 0.5 mm CaCl_2_, 2 mm MgCl_2_, 10 mm HEPES, 2 mm MgATP, and 0.1 mm GTP; pH adjusted to 7.25 with Tris base; osmolarity adjusted to 290 mOsm with sucrose. The internal solution contained QX314 (2 mm) for improved voltage control.

### Standard patch-clamp electrophysiology

Electrophysiology experiments were conducted using a Nikon Eclipse FN-1 upright microscope equipped with a 40× (0.8 NA) water-dipping (3.3-mm working distance) objective. Neurons within brain slices were first visualized with infrared or visible differential interference contrast (DIC) optics. A computer running pCLAMP 10 software was used to acquire whole-cell recordings along with a Multiclamp 700B amplifier and a Digidata 1550A A/D converter (all from Molecular Devices Inc.). Data were sampled at 10 kHz and low-pass filtered at 1 kHz. Immediately before giga seal formation, the junction potential between the patch pipette and the superfusion medium was nulled. Series resistance was uncompensated. To record physiological events following local application of drugs, a drug-filled pipette was moved to within 20–40 μm of the recorded neuron using a second micromanipulator. A Picospritzer (General Valve) dispensed drug (dissolved in recording solution) onto the recorded neuron via a pressure ejection. Pipette location relative to the recorded cell, along with ejection pressure, were held constant throughout the recording. Ejection duration was varied at half-log steps to enable collection of quasi-concentration response curves. For standard patch-clamp recordings, number of rats used were as follows: *n* = 5 (nicotine SA), *n* = 3 (yoked saline), *n* = 3 (saline SA).

### 2PLSM, electrophysiology, and nicotine uncaging

Photoactivatable nicotine (PA-Nic) photolysis was performed as previously described (Banala et al., 2018; Yan et al., 2018; Arvin et al., 2019a,b). A modified Olympus BX51 upright microscope and a 60× (1.0 NA) water-dipping (2-mm working distance) objective was used to visualize cells. Prairie View 5.5 (Bruker Nano) software was used for image acquisition, photostimulation, and electrophysiology acquisition via a Multiclamp 700B patch-clamp amplifier. Analog signals were sampled at 1 kHz and an A/D converter (6052; National Instruments) was used for digitization. Patch-clamp recordings were conducted using the internal solution mentioned above, except that Alexa Fluor 488 (hydrazide salt; 100 μm) was also included in the recording pipette to visualize cells using 2PLSM. After break-in, the internal solution with the Alexa Fluor dye was allowed to equilibrate for 15–20 min before imaging was initiated. A Chameleon Ultra I (Coherent Laser Group) tunable (690–1040 nm) Ti:sapphire laser system tuned to 930 nm (80-MHz pulse repetition frequency and ∼140-fs pulse duration) was used to excite Alexa Fluor 488. A M350-80-02-BK Pockels cell (ConOptics) was used for power attenuation. The system was equipped with two non-de-scanned detectors (Hamamatsu side-on multi-alkali R3896 photomultiplier tubes) for detection of green and red wavelengths (emission filters: 525/70 nm, 595/50 nm), but only the green channel was used in this study. A 405-nm continuous wave laser (100-mW OBIS LX; Coherent) was used for photostimulation/uncaging via a partially independent light path and a second set of *x-y* galvanometers incorporated into the scanhead (Cambridge Technologies). Laser power from the imaging and uncaging beams was measured as the beam exited the scanhead at the turndown mirror position, which is above the primary dichroic and microscope objective. An additional ∼20% loss of power is expected between the point where we measured the power and the sample. Power was measured using an integrating sphere photodiode power sensor (S142C; Thorlabs). PA-Nic (50 μm) was dissolved in 10 ml of recording solution and the solution was applied to the slice via a recirculation system. The Markpoints module of Prairie View 5.5 software was used to select spots in the field of view (∼1 μm in diameter) for focal uncaging of nicotine via 405-nm laser light flashes (15 or 50 ms, 3–4 mW). For some recorded cells, a Z-series 2PLSM image of the cellular morphology was acquired after completion of electrophysiological recordings. A maximum intensity projection from such Z-series images was used to display uncaging positions along dendrites. For 2PLSM recordings, number of rats used were as follows: *n* = 3 (nicotine SA), *n* = 4 (saline SA).

### Experimental design and statistical analysis

SA data produced by Med Associates MedPC IV software were ingested, processed, and graphed with custom scripts written in MATLAB (MathWorks). Some SA data was analyzed/graphed with GraphPad Prism 7 software. Electrophysiology data files produced by pClamp or Prairie View software were ingested and processed with custom MATLAB scripts and Origin (OriginLab) software. 2PLSM image data were analyzed and processed with ImageJ. Heat maps of electrophysiology traces were produced in MATLAB using the cubeYF colormap (Matteo Niccoli; Perceptually improved colormaps). Scalable vector graphics were produced in MATLAB using functions written by Salva Ardid (https://github.com/kupiqu/fig2svg). Rat brain anatomy graphics were derived from “Brain Maps 4.0” (Larry Swanson; University of Southern California; Swanson, 2018).

For statistical analysis, the following effect sizes were used/calculated: mean difference (for SA data) and median difference (for electrophysiology data). Two parallel approaches were used for statistical analysis: null hypothesis testing and confidence interval (CI) estimation. Under null hypothesis testing, the two groups being compared are assumed not to differ (the mean difference or median difference is zero) and are pooled. A total of 5000 permutations were constructed, where each permutation is a re-ordering of the original pooled data into two groups. The mean (or median) difference is calculated for each re-ordering. The reported *p* value represents the likelihood of observing a mean (or median) difference greater than or equal to the one we report if the null hypothesis is true. Under the estimation approach (Calin-Jageman and Cumming, 2019), the null hypothesis of no difference is not assumed and the groups are permitted to differ. A bootstrap approach was used whereby 5000 bootstrap resamples, with replacement, were taken. The bootstrap sampling distribution and the 95% CIs for the mean or median differences were determined in R using the ‘dabestr’ package, and Gardner–Altman estimation plots (Ho et al., 2019) were produced with assistance from the following web app (https://www.estimationstats.com/).

## Results

### Nicotine SA

**Figure 1.**
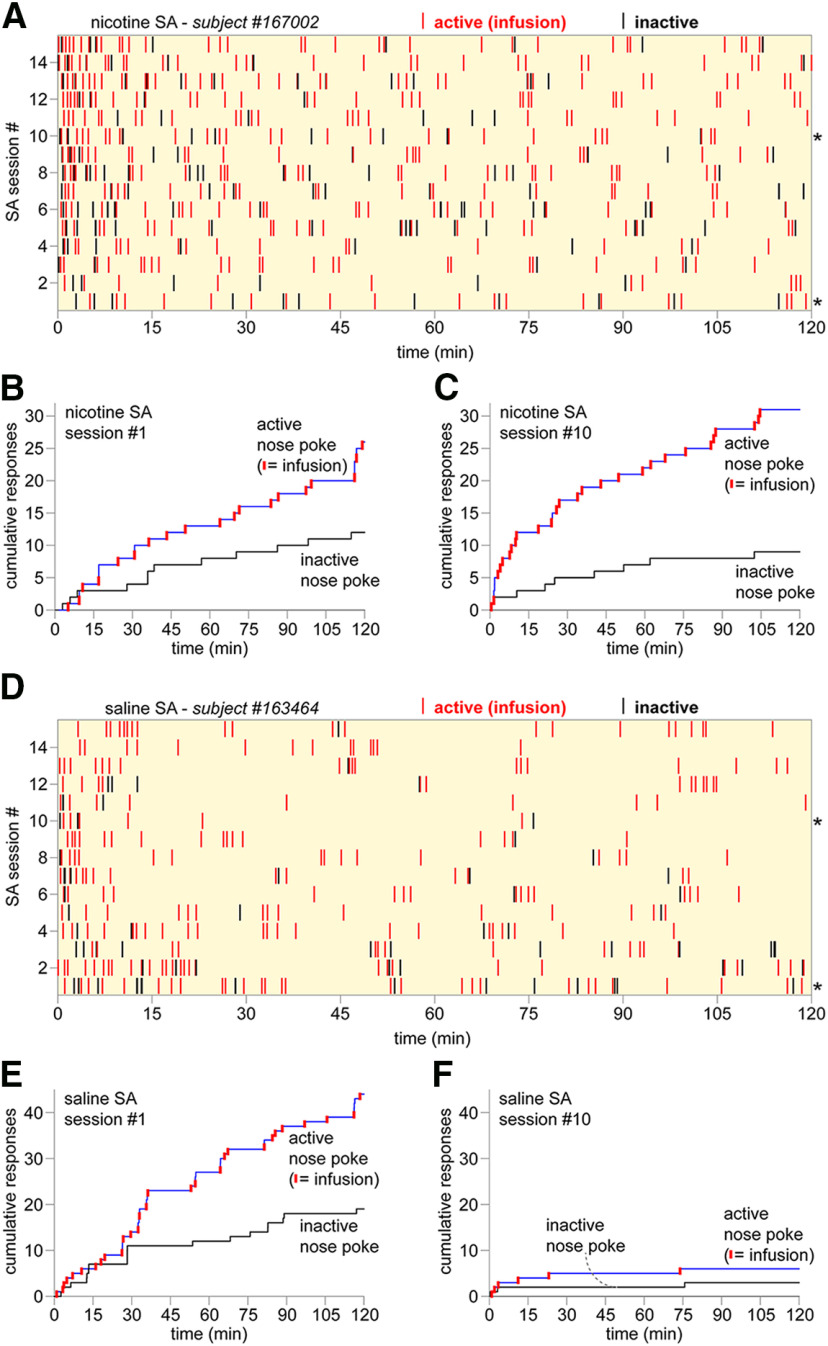
Nicotine SA representative results. ***A***, Nicotine SA training history for rat #167002 is shown as a raster plot. Each nose poke response (active resulting in an infusion: red; inactive: black) during the 2-h SA session is represented as a vertical tick mark. SA sessions, from first to 15th, are plotted from bottom to top; *denotes sessions plotted in further detail in ***B***, ***C***. ***B***, ***C***, Cumulative response plot for session #1 (***B***) and #10 (***C***) are shown for nicotine SA rat #167002. Active nose pokes that occurred during the timeout period, which did not result in a nicotine infusion, are shown as upward-going blue tick marks. ***D***, Saline SA training history for rat #163464 is shown as a raster plot. Each nose poke response (active resulting in an infusion: red; inactive: black) during the 2-h SA session is represented as a vertical tick mark. SA sessions, from first to 15th, are plotted from bottom to top; *denotes sessions plotted in further detail in ***E***, ***F***. ***E***, ***F***, Cumulative response plot for session #1 (***E***) and #10 (***F***) are shown for nicotine SA rat #163464. Active nose pokes that occurred during the timeout period, which did not result in a saline infusion, are shown as upward-going blue tick marks.

To examine nAChR functional upregulation in the context of volitional nicotine intake, we first established a rat model of intravenous nicotine SA. Male Sprague Dawley rats were implanted with indwelling jugular catheters and trained to nose poke for an infusion of nicotine (0.03 mg/kg/infusion; 2-h sessions) on a FR1 schedule of reinforcement. Operant chambers were equipped with an active nose poke (paired with nicotine) and an inactive nose poke where a response had no consequence. Training involved up to 15 sessions. A typical nicotine SA training history for a single rat is shown (Fig. 1*A*), including active and inactive responses. Cumulative response plots (active and inactive responses) are shown for the same rat on days 1 and 10 of nicotine SA training (Fig. 1*B*,*C*). A separate group of rats were trained to nose poke for a saline (vehicle) infusion. When compared with nicotine SA, this control isolates the animal’s interest in the visual cues rather than the infused drug. Examination of the training history (Fig. 1*D*) for a representative rat, as well as the cumulative response plots (Fig. 1*E*,*F*), suggests that initial (day 1) responding may be driven primarily by novelty seeking. As novelty fades (i.e., day 10), saline does not maintain robust responding whereas nicotine does (Fig. 1*C* vs *F*).

**Figure 2.**
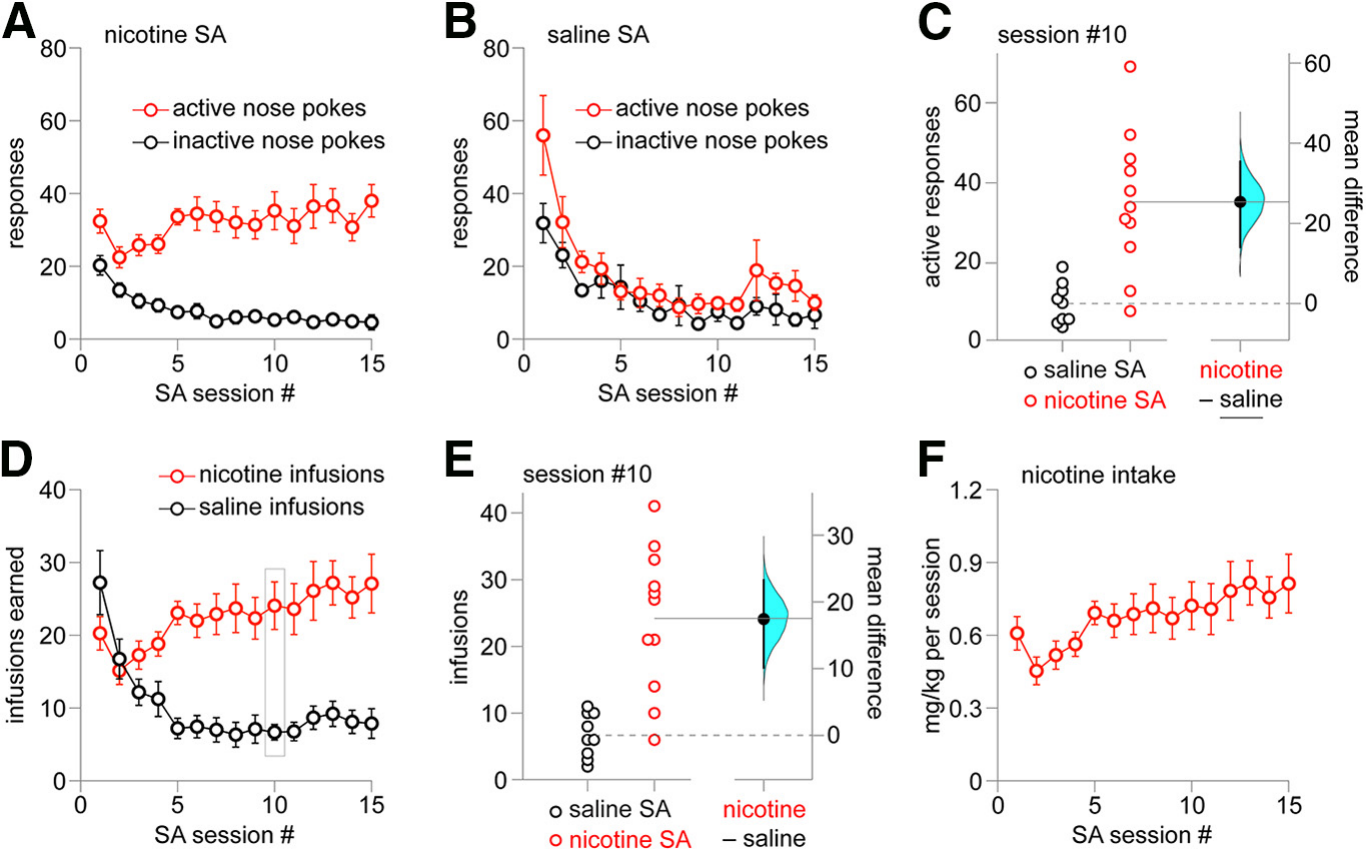
Nicotine SA summary results. ***A***, Nicotine SA responses. Mean (±SEM) active and inactive nose pokes for *n* = 15 male rats are shown for sessions #1–15 of nicotine SA. ***B***, Saline SA responses. Mean (±SEM) active and inactive nose pokes for *n* = 12 male rats are shown for sessions #1–15 of saline SA. ***C***, Gardner–Altman plot of active nose poke responses on SA session #10 for nicotine SA and saline SA groups. The effect size (mean difference) and bootstrap 95% CI are shown at right. ***D***, Nicotine versus saline infusions. Mean (±SEM) # of nicotine and saline infusions earned are shown for SA sessions #1–15. ***E***, Gardner–Altman plot of infusions earned on SA session #10 for nicotine SA and saline SA groups. The effect size (mean difference) and bootstrap 95% CI are shown at right. ***F***, Nicotine intake. Mean (±SEM) nicotine intake is shown for the nicotine SA group for SA sessions #1–15.

Summary active/inactive nose poke response data are shown for 15 d of training in the nicotine SA (Fig. 2*A*) and saline SA (Fig. 2*B*) group. For nicotine SA on day 10 of training, the mean difference between active and inactive responses is 30.0 (bootstrap 95% CI: 20.1, 40.3). For saline SA on day 10 of training, the mean difference between active and inactive responses is 2.33 (bootstrap 95% CI: −5.67, 6.89). A Gardner–Altman plot (Fig. 2*C*) shows active responses for individual rats on day 10 of nicotine or saline training, with mean difference (25.4 active responses) and bootstrap 95% CI (14.1, 35.4) shown on the floating axes on the right. A two-sided permutation test (*p *=* *0.0008) indicated that it is very unlikely to observe this effect size if the saline SA and nicotine SA groups do not differ. For saline SA, responding decreased to a stable level of ∼10 responses on each nose poke after initial interest on the first 3–5 d (Fig. 2*C*). Examination of infusions earned in the nicotine versus saline SA group showed substantially different patterns for saline versus nicotine, with nicotine infusions supporting stable responding (∼20–30 infusions/session) from day ∼5 onward (Fig. 2*D*). A Gardner–Altman plot (Fig. 2*E*) shows saline versus nicotine infusions for individual rats on day 10 of training, with mean difference (17.4 infusions) and bootstrap 95% CI (10.1, 23.3) shown on the floating axes on the right. A two-sided permutation test (*p *=* *0.0006) indicated that it is very unlikely to observe this effect size if the saline SA and nicotine SA groups do not differ. Summary data for total nicotine intake during each 2-h session were calculated for the nicotine SA group (Fig. 2*F*). Next, we examined nAChR functional plasticity in MHb, a brain region critical to nicotine dependence.

### Functional nAChR upregulation

**Figure 3.**
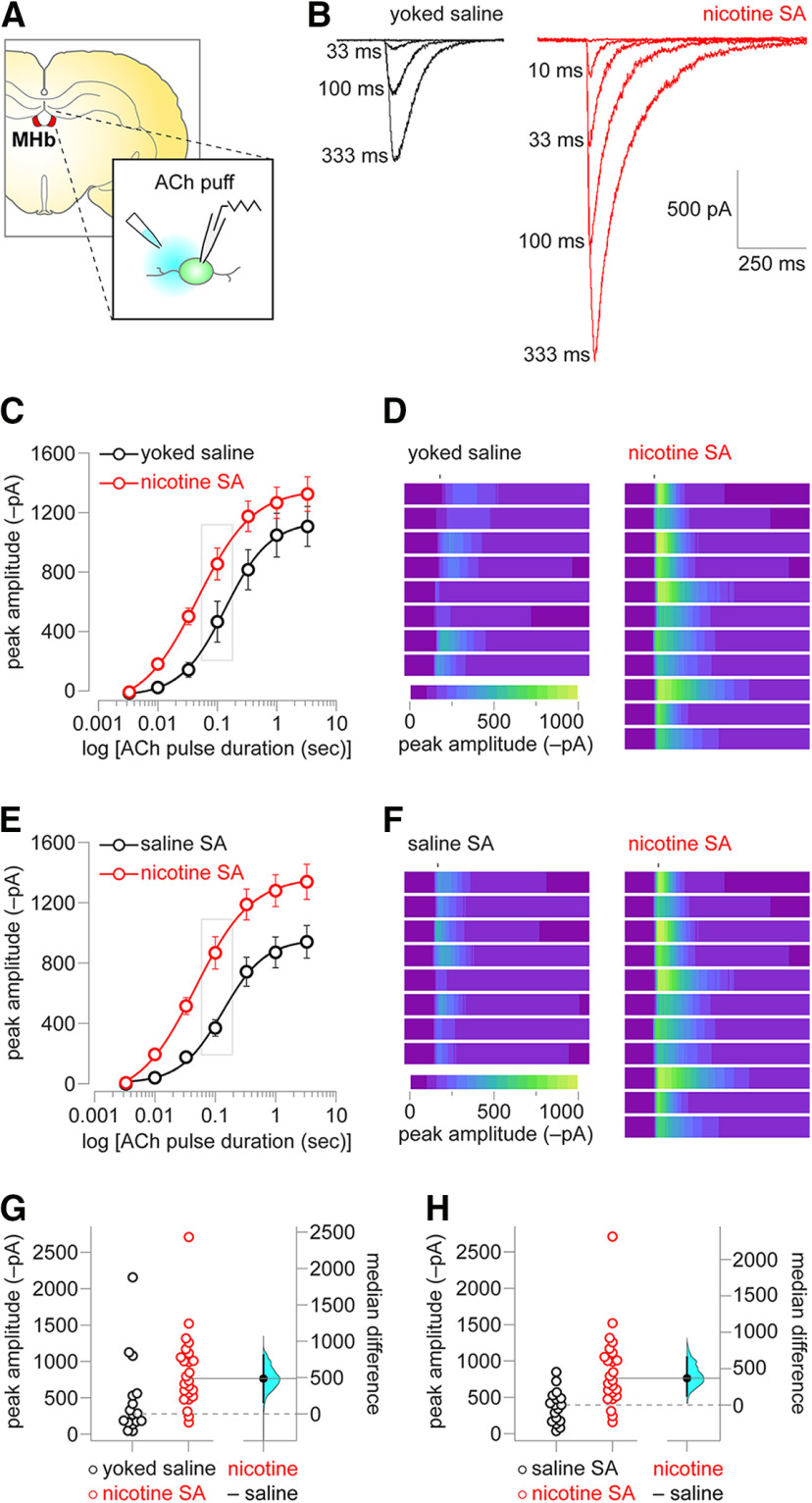
nAChR functional upregulation in MHb. ***A***, Experiment schematic. Coronal brain slices containing MHb were prepared from rats after the last SA session. MHb neurons were patch clamped and ACh was locally applied to the neuron to evoke nAChR currents. ***B***, nAChR representative responses in nicotine SA versus yoked saline rats. Responses from the same MHb neuron to the indicated ACh pulse duration are shown for nicotine SA or yoked saline control. ***C***, Summary concentration response data for nicotine SA versus yoked saline. Mean (±SEM) ACh-evoked current amplitude is shown for the indicated ACh pulse duration for recordings from MHb neurons from nicotine SA and yoked saline control rats. ***D***, Heat map representation of ACh-evoked currents. For 100-ms ACh pulses in the nicotine SA and yoked saline control groups, individual cellular responses comprising the second and third quartiles are expressed as a heat map scaled to the response with the greatest magnitude (a nicotine SA response). ***E***, Summary concentration response data for nicotine SA versus saline SA. Mean (±SEM) ACh-evoked current amplitude is shown for the indicated ACh pulse duration for recordings from MHb neurons from nicotine SA and saline SA rats. Nicotine SA data from ***C*** are re-plotted for comparison to the saline SA control group. ***F***, Heat map representation of ACh-evoked currents. For 100-ms ACh pulses in the nicotine SA and saline SA groups, individual cellular responses comprising the second and third quartiles are expressed as a heat map scaled to the response with the greatest magnitude (a nicotine SA response). ***G***, Gardner–Altman plot of peak ACh-evoked current (100-ms pulse duration) for nicotine SA versus yoked saline control. The effect size (median difference) and bootstrap 95% CI are shown at right. ***H***, Gardner–Altman plot of peak ACh-evoked current (100-ms pulse duration) for nicotine SA versus saline SA. The effect size (median difference) and bootstrap 95% CI are shown at right.

**Figure 4.**
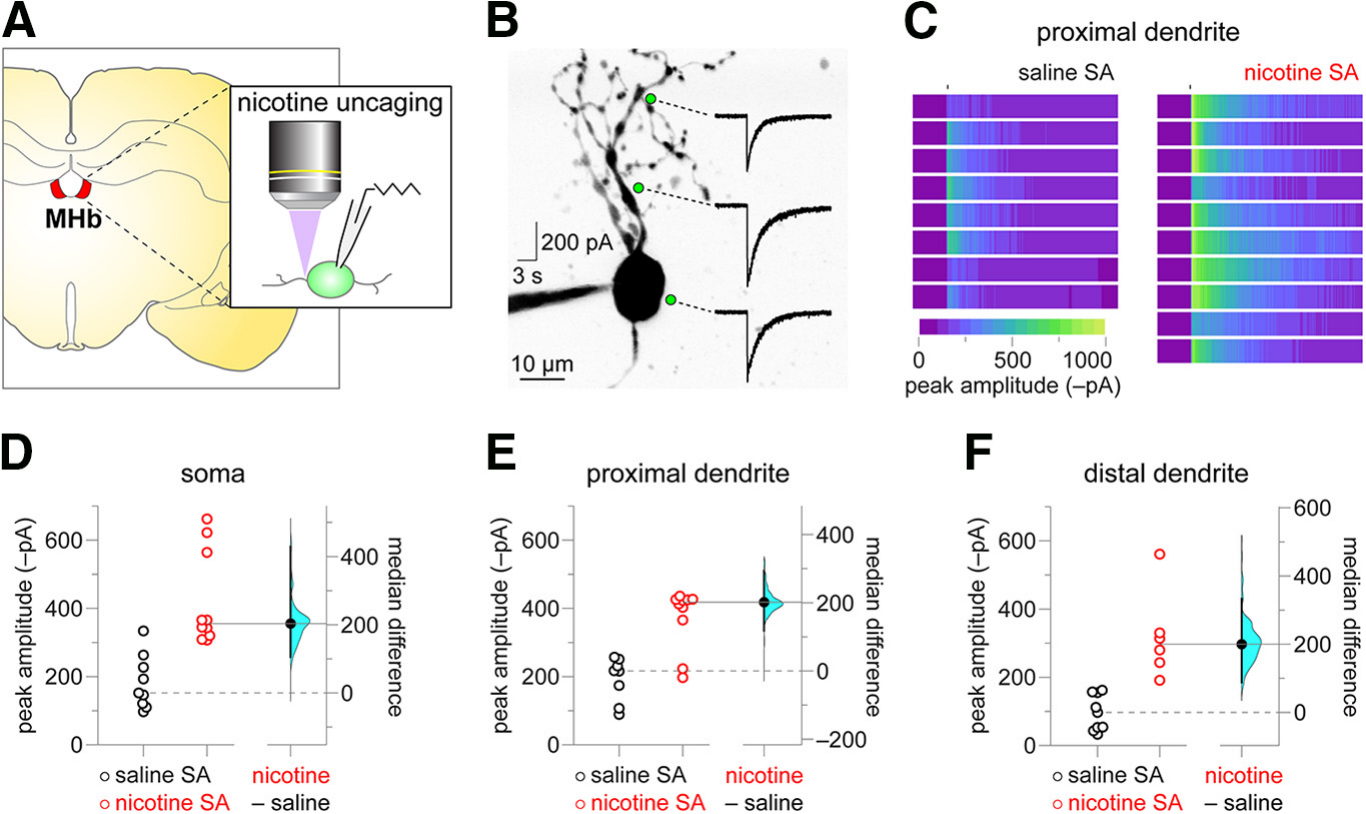
Nicotine SA boosts nAChR function at the soma and in dendrites. ***A***, Experiment schematic. Coronal brain slices containing MHb were prepared from rats after the last SA session. MHb neurons were patch clamped, neuronal morphology was imaged with 2PLSM, and nicotine was uncaged via laser flash photolysis. ***B***, Representative nicotine uncaging experiment. An exemplar two-photon image of a MHb neuron is shown, along with locations (soma, proximal dendrite, distal dendrite) where nicotine was uncaged and the recorded nAChR response to such uncaging. ***C***, Proximal dendrite uncaging responses. Heat map representations of proximal dendrite nicotine uncaging responses from individual neurons are shown for nicotine SA and saline SA rats. All heat maps are scaled to the response with the greatest magnitude (a nicotine SA response). ***D–F***, Gardner–Altman plots of peak nicotine uncaging currents at the soma (***D***), proximal dendrite (***E***), and distal dendrite (***F***). At left, scatter plots show individual uncaging currents for nicotine SA and saline SA rats. At right, the effect size (median difference) and bootstrap 95% CI are shown.

After 15 d of operant training, rats were killed and *ex vivo* brain slices were prepared for patch-clamp recordings. Coronal slices were cut to include the MHb, a small epithalamic structure immediately ventral to the hippocampus and adjacent to the third ventricle (Fig. 3*A*). Two control groups were prepared: saline SA rats as described above, and a yoked saline group. The latter was prepared by implanting indwelling jugular catheters and administering rats a number of non-contingent saline infusions. Whole-cell patch-clamp recordings were made from MHb neurons in the ventral inferior subnucleus (Aizawa et al., 2012), which are expected to be dual cholinergic/glutamatergic (Ren et al., 2011) and positive for nAChR expression based on our prior work (Shih et al., 2015). ACh was locally applied to the recorded neuron to elicit nAChR activation. The location of the pipette tip was held constant, whereas the duration of the pressure ejection was varied, allowing for a quasi-concentration response curve to be generated. A representative trace family from a MHb neuron from the yoked saline and nicotine SA group is shown (Fig. 3*B*), illustrating that nicotine SA induced a substantial increase in nAChR function. Summary data (ACh pulse duration vs peak inward current) for yoked saline versus nicotine SA indicates a leftward and an upward shift in the relationship between ACh pulse duration (which should be highly correlated with the ACh concentration delivered to the cell) and peak inward current amplitude. To further illustrate the effect of nicotine SA on nAChR function, all individual responses to a 100-ms ACh pulse in the second and third quartile of the yoked saline and nicotine SA datasets are expressed as a heat map scaled to the largest response in the nicotine SA group (Fig. 3*D*). The same nicotine SA dataset was also compared with the dataset taken from saline SA rats. A very similar leftward/upward shift was seen in the relationship between ACh pulse duration and peak current (Fig. 3*E*). Likewise, heat map representation of second and third quartile responses to 100-ms ACh demonstrate a substantial enhancement in nAChR function in the nicotine SA group (Fig. 3*F*). A Gardner–Altman plot (Fig. 3*G*) is shown for ACh response amplitude (100-ms pulse duration) in the yoked saline versus nicotine SA groups, with median difference (486 pA) and bootstrap 95% CI (162, 809) shown on the floating axes on the right. A two-sided permutation test (*p *=* *0.0112) indicated that it is unlikely to observe this effect size if the yoked saline and nicotine SA groups do not differ. A Gardner–Altman plot (Fig. 3*H*) is shown for ACh response amplitude (100-ms pulse duration) in the saline SA versus nicotine SA groups, with median difference (368 pA) and bootstrap 95% CI (126, 658) shown on the floating axes on the right. A two-sided permutation test (*p *=* *0.0052) indicated that it is unlikely to observe this effect size if the saline SA and nicotine SA groups do not differ. These data demonstrate substantial nAChR functional enhancement in MHb neurons following acquisition of nicotine SA behavior. Next, we examined this enhancement at different neuronal locations using nicotine uncaging and 2PLSM (Fig. 4*A*).

We previously demonstrated that chronic passive nicotine exposure induced nAChR functional upregulation at MHb perisomatic, proximal, and distal dendrite locations using nicotine uncaging and 2PLSM (Banala et al., 2018). Here, we asked whether such nAChR functional plasticity was similarly induced by nicotine SA. Slices were prepared from nicotine SA or saline SA rats, and MHb neuronal morphology was visualized via 2PLSM by filling neurons with Alexa Fluor 488 during whole-cell patch-clamp recordings. PA-Nic was bath-applied to the slice, followed by photolysis with focal delivery of 405-nm light pulses. These pulses were delivered to any desired location relative to the imaged neuron via a continuous wave 405-nm laser connected to a dedicated light path and pair of *x-y* galvanometers. The following spatial locations were chosen for nicotine uncaging for each recorded/imaged neuron: (1) perisomatic, (2) on a primary dendrite 10–15 μm from the soma (proximal dendrite), and (3) on a primary dendrite 30–45 μm from the soma (distal dendrite). A representative maximum intensity projection image of a MHb neuron, along with nicotine uncaging responses at these three locations, is shown (Fig. 4*B*). Compared with uncaging responses in saline SA MHb neurons, uncaging responses in MHb neurons from nicotine SA rats were more substantial. To illustrate this, heat map representations of all saline SA and nicotine SA responses at the proximal dendrite are shown scaled to the largest response in the nicotine SA group (Fig. 4*C*). Gardner–Altman plots are shown for nicotine uncaging peak current amplitude in the saline SA versus nicotine SA groups at the soma (Fig. 4*D*), proximal dendrite (Fig. 4*E*), and distal dendrite (Fig. 4*F*). Median difference and bootstrap 95% CI for these comparisons are 204 (106, 429) pA, 202 (118, 294) pA, and 202 (88, 334) pA, respectively. Two-sided permutation tests (*p *=* *0.0, 0.0058, and 0.0, respectively) indicated that it is very unlikely to observe these effect sizes if the saline SA and nicotine SA groups do not differ. These data demonstrate that nicotine SA induces functional upregulation of nAChRs in MHb neurons at the soma and along the dendritic tree.

## Discussion

In this study, we established intravenous nicotine SA and examined its effect on nAChR function and cellular distribution in MHb neurons. We report that nAChR function on the surface of MHb neurons is substantially enhanced by nicotine SA. Upregulation is evident on the soma cell membrane and on proximal and distal dendrites. By examining nAChR functional activity in neurons from rats with a history of nicotine SA, this study adds new mechanistic information related to nicotine dependence.

### Nicotine SA

Corrigall and colleagues demonstrated the first robust model of FR nicotine SA in rat (Corrigall and Coen, 1989), including the importance of dopaminergic centers on nicotine reinforcement (Corrigall and Coen, 1991; Corrigall et al., 1994). Subsequently, Donny and colleagues validated and extended this model (Donny et al., 1995), probing important issues such as motivation (Donny et al., 1999) and cue dependency (Caggiula et al., 2001). Many other laboratories have successfully implemented this technique, leading to important translational insights into nicotine’s neuropharmacological actions. Our nicotine SA model is based on the principles and design parameters first elucidated by Corrigall and Donny, which yielded consistent results.

Consistent with prior work (Clemens et al., 2010; Caille et al., 2012), we noted a substantial increase in initial responding for nicotine when rats were briefly trained to nose poke for food pellets compared with no prior food training (data not shown). Drug SA studies in rat have typically used levers as the operandum, but nose pokes promote naturalistic exploratory behavior and may better enable acquisition of nicotine SA (Clemens et al., 2010). Our SA session parameters were similar or identical to a prior nicotine SA study using nose pokes, and we observed very similar nicotine and saline SA active/inactive responses, nicotine/saline infusions, and total nicotine intake (Clemens et al., 2010). Nicotine intake (0.6–0.8 mg/kg in 2 h) in our model was also similar to previous pioneering studies, where an intake rate of roughly 0.5 mg/kg/h was observed (Corrigall and Coen, 1989; Donny et al., 1995; Fowler et al., 2011). Our results may show a slightly lower total nicotine intake because of the 2-h session duration; training history raster plots (Fig. 1*A*) and cumulative response plots (Fig. 1*C*) suggest that for well-trained rats, a majority of the responding occurs within the first h of the session. This is consistent with the development of tolerance to nicotine following repeated exposure, leading to “loading” and “maintenance” behavior similar to what is observed in human smokers (Benowitz, 1992). Overall, these data confirm that we successfully established a rat model of nicotine SA.

### nAChR functional upregulation

We previously demonstrated nAChR upregulation in MHb in mice using non-contingent nicotine exposure via osmotic minipumps (Shih et al., 2015) or nicotine delivered in the drinking water (Banala et al., 2018). Tapper and colleagues also reported similar findings (Pang et al., 2016), where mouse nAChRs potentially containing α6 subunits were upregulated by chronic, passive nicotine exposure. Given our prior results showing that α6 subunits are preferentially expressed in the MHb subnucleus where we performed our recordings in the present study (Shih et al., 2014, 2015), α6-containing nAChRs are a possible candidate subtype that is upregulated by nicotine SA. α3β4 nAChRs are robustly expressed throughout the ventral MHb and account for ≥50% of agonist-induced current responses (Quick et al., 1999; Shih et al., 2014, 2015; Pang et al., 2016), suggesting a contribution by these receptors to upregulation induced by SA. Such receptors have been known to upregulate in response to nicotine (Mazzo et al., 2013; but also see Wang et al., 1998).

Because (1) mice metabolize nicotine much faster than rats (Matta et al., 2007) and (2) contingent nicotine is known to provoke changes to receptors and circuits that non-contingent nicotine does not (Caillé et al., 2009; Metaxas et al., 2010), the outcome of the experiments we report here were not obvious. Importantly, the degree of functional enhancement we observed in our rat nicotine SA model is consistent with our previous studies that used patch-clamp electrophysiology. nAChR functional enhancement is most apparent for ACh pulses of short (10–100 ms) duration (Fig. 3*C*,*E*). Given the brief lifetime of ACh at nAChRs because of rapid degradation by acetylcholinesterase (Wilson and Harrison, 1961), our data indicate that transmission of physiological cholinergic signals is very likely to be enhanced by nAChR upregulation. Our use of yoked saline and saline SA control groups helps us draw the conclusion that the conditioned cues associated with operant responding cannot account for nAChR upregulation. nAChR function in our assays is unlikely to be attenuated by acute desensitization from residual nicotine, as rats had completed their last nicotine SA session ∼24 h before slice preparation. Given that the *t*_1/2_ of nicotine in rats is ∼45 min (Matta et al., 2007), and that nicotine is cleared from neurons very rapidly once exposure stops (Shivange et al., 2019), the amount of residual nicotine to which neurons would be exposed in our recordings is negligible.

PA-Nic photolysis with focal 405 nm laser pulses enabled us to examine the cellular distribution of upregulated nAChRs in non-contingent nicotine exposure models (Banala et al., 2018), as well as identify cellular sites of functional nAChR expression in ventral tegmental area glutamate neurons for the first time (Yan et al., 2018). In the present study, we applied this approach to the question of whether nicotine exposure via SA induces nAChR upregulation in specific cellular locations or generally throughout the cell. Our data (Fig. 4) are more consistent with the latter conclusion, as we observed enhanced nAChR function at the soma, proximal dendrites, and distal dendrites of MHb neurons from rats with a history of nicotine SA. We previously measured the lateral distance at half-maximum amplitude for nicotine uncaging to be 4.5 μm, and a distance of ∼10 μm is expected to elicit a weak but still measurable response (Banala et al., 2018). In the present study, the lateral distance between our somatic and proximal dendrite uncaging positions is 10–15 μm, suggesting a very minor contribution of somatic nAChRs to proximal dendrite responses (and vice versa). With this context, the lateral ∼30-μm distance between our proximal versus distal dendrite uncaging locations suggests little to no cross-activation of these receptor populations.

Our 2PLSM-assisted uncaging results (Fig. 4), along with consistent previous uncaging data from mice exposed passively to nicotine (Banala et al., 2018), indicate that nicotine exposure induces a process that deposits additional nAChRs on the membrane in a non-specific manner. A reasonable inference from this is that nicotine exposure simply amplifies what is a generally non-specific biosynthetic trafficking process. This is consistent with imaging studies of nAChRs expressed at physiological levels in native or cultured neurons (Nashmi et al., 2007; Mackey et al., 2012), where the receptors appear diffusely distributed throughout the interior of the cell and non-specifically on the cell membrane.

### The habenulo-interpeduncular system

The MHb and its target the interpeduncular nucleus form a relay system linking forebrain structures (i.e., septal nuclei) with midbrain/hindbrain limbic centers such as the median raphe, the pons, and possibly the ventral tegmental area (Qin and Luo, 2009; Hsu et al., 2013; Gardon et al., 2014; Zhao-Shea et al., 2015; Quina et al., 2017; Morton et al., 2018; Wolfman et al., 2018). The ventral two thirds of the MHb are dual-transmitting cholinergic/glutamatergic neurons (Ren et al., 2011; Aizawa et al., 2012) with exceptionally high levels of nAChRs that are primarily low-sensitivity β4 nAChR subunit-containing receptors (Quick et al., 1999; Shih et al., 2014, 2015). Global knock-out or MHb knock-down of α5 nAChR subunits, which may be components of MHb nAChRs (Marks et al., 1992; Frahm et al., 2011), was associated with diminished sensitivity to the aversive aspect of high nicotine doses without impacting nicotine SA of low, rewarding doses (Fowler et al., 2011). These results, along with others (Salas et al., 2004, 2009; Frahm et al., 2011; Zhao-Shea et al., 2013), have supported the inference that MHb nAChRs are involved primarily in withdrawal and other behavioral responses triggered by aversive nicotine concentrations. It is therefore curious that we observed strong functional upregulation of MHb nAChRs following a relatively modest nicotine exposure (∼0.75 mg/kg/d) using 2-h, 5-d/week SA sessions. These results suggest that simply acquiring nicotine SA leads to modulation of nAChRs in systems such as MHb that are linked more prominently to nicotine dependence rather than reward. Future additional studies comparing responses in yoked nicotine versus nicotine SA rats would help substantiate this idea. Our results suggest that acquiring nicotine SA induces plastic changes that prime cholinergic brains areas in ways that may make the individual more susceptible to withdrawal and relapse.

### Conclusions and future directions

This study reports an electrophysiological examination of nAChR function in neurons from rats which self-administered nicotine. We speculate that the rarity of such reports is because of the technical challenge of combining nicotine SA behavior with patch-clamp recordings from adult rat brain slices (however, see Caillé et al., 2009; Gipson et al., 2013). The present study highlights the importance of making functional measurements in valid preclinical models of substance abuse. For example, functional interrogation of the dopamine transporter in tissue from rats who had SA cocaine has yielded important translational insights into the induction of a hypodopaminergic state by repeated psychostimulant use (Calipari et al., 2013; Siciliano et al., 2016). We expect future studies of nAChR sensitization in nicotine SA models, perhaps including those with longer nicotine access and/or those that probe relapse, to similarly contribute to the nicotine and tobacco dependence literature.

## Synthesis

Reviewing Editor: Yavin Shaham, NIDA-IRP/NIH Decisions are customarily a result of the Reviewing Editor and the peer reviewers coming together and discussing their recommendations until a consensus is reached. When revisions are invited, a fact-based synthesis statement explaining their decision and outlining what is needed to prepare a revision will be listed below. The following reviewer(s) agreed to reveal their identity: Cassandra Gipson.

The reviewers found significant merit in the study but have made several comments that should be addressed in the revision. Based on their evaluation, I do not think that additional experimental data are necessary for a successful revision. I enclose below the individual comments of the reviewers that should be addressed with text changes. In addition to their comments, please address the comments below:

1. Abstract and other places in the text: please change the 1st sentence to rephrase the information as a hypothesis, not a fact. Also change ‘contingent nicotine delivery’ to ‘nicotine self-administration’ and ‘To our knowledge, this study is the first to demonstrate’ to ‘Our study demonstrates.’

2. Methods and other places in the text: please change ‘animals’ to ‘rats’ when referring to the experimental subjects.

3. Discussion: please change ‘To our knowledge, we are the first to report’ to ‘We report’ (both in the beginning and the end of this section)

4. Figures, figure legends and other places in the text: please spell out NIC to nicotine and NP to nosepoke. In general, avoid using uncommon abbreviations as these make it more difficult to read the paper. I also recommend increasing the font size of some of the figures to size 8 or 9 to improve readability.

REVIEWER 1

This is an interesting manuscript describing functional upregulation of nicotinic acetylcholine receptors (nAChRs) in the medial habenula (MHb) after rats acquire nicotine operant self-administration. Importantly, functional upregulation of nAChRs is hypothesized to be a critical neuro-adaptation to chronic nicotine exposure associated with nicotine withdrawal symptoms, craving, and/or dependence. In addition, the MHb is a brain area emerging as critical for nicotine intake and withdrawal in part due to the dense expression of nAChRs in the ventral portion of the nucleus. While previous work by the Drenan lab and others (including Pang et al., 2016 Neuropharmacology, which should be cited), indicate passive nicotine functionally upregulates MHb nAChRs, whether operant nicotine self-administration is sufficient to upregulate nAChRs is unknown. Jin et al., bridge this knowledge gap by demonstrating that rats trained to nose-poke for nicotine, after acquisition and maintenance of nicotine self-administration, exhibit upregulated responses to acetylcholine in MHb slices compared to yoked saline groups. In addition, the authors use caged nicotine to probe nAChR upregulation in sub-cellular locations of MHb neurons. Overall, the manuscript is well written, the experiments technically sound, and the data described should be of value to those in the field. I have relatively minor comments/discussion points that should be considered.

1. The location and sub-population(s) of MHb neuron that were recorded is not indicated. It would be helpful to know if the neurons undergoing upregulation were cholinergic or at least in the dorsal or ventral portion of the MHb as these are functionally distinct. Also, a discussion of potential differences in upregulation between sub-populations or nAChR sub-types would be informative.

2. On page 21 of the discussion, the authors state that it is curious that relatively low nicotine exposure via self-administration elicits such robust upregulation. They also make the point that it is critical to measure plasticity elicited by contingent nicotine exposure as opposed to passive exposure. Although not explicitly stated, the implication is that the behavior of self-administering drug in combination with the drug itself may have a more robust or distinct effect compared to passive drug administration. Thus, it is somewhat of a missed opportunity to not include a yoked nicotine group to test if rats that receive the exact amount of nicotine as self-administering animals but without control of exposure also exhibit functionally upregulated nAChRs in the MHb. This control experiment would be necessary before concluding that “simply acquiring nicotine SA leads to modulation of nAChRs...” The authors should consider including this point in their discussion.

REVIEWER 2

The manuscript titled “Nicotine self-administration induces plastic changes to nicotinic receptors in medial habenula” is a well-written, rigorously conducted series of experiments in which the functional changes in nAChRs within this key region heavily implicated in nicotine dependence are characterized. Overall, these studies show enhancement of nAChR function in various locations of cells including the soma, proximal, and distal dendrites using cutting edge techniques such as whole cell patch clamp, 2-photon laser scanning microscopy, and nicotine uncaging using photoactivatable nicotine. These techniques allow unprecedented understanding of nAChR functional changes relevant to nicotine self-administration. The manuscript includes nicotine self-administering, saline self-administering, and yoked saline groups, enhancing rigor. Overall, there is great enthusiasm for this manuscript. Minor issues are listed below:

1. The nicotine infusion duration is quite short, being 2-sec in duration. What was the justification for delivery of drug over this short of a period of time?

2. The methods state that animals were able to self-administer for up to 15 sessions, with stabilization around 5-7 days. The subsequent sentence claims that an animal was dropped from the study due to lack of acquisition. This is confusing, as it is unclear whether there were acquisition criteria, or if animals were simply run to 15 sessions regardless of performance. Please clarify, and justify this methodology.

3. Related to the point above, what was considered “stable responding”?

4. Only 3 animals were included in the yoked saline group. This is a very low n, regardless of the fact that this group was used for the electrophysiology studies.

5. It is unclear why individual rat data are included in Figure 1, but only for one rat per group. If individual data are to be presented, it only makes sense to do so for each animal, as individual differences in self-administration, especially with an outbred strain (Sprague Dawley), likely makes these representative animals not representative of the groups. It is recommended that Figure 1 be omitted given these concerns (to support this point, data in Figure 2E show large individual differences in infusions by session 10, which is presumably after stability given the 5-7 day range that is stated in the manuscript).

6. The results section contains duplicative methodology. Please omit this information from the results section.

7. It is unclear what type of neurons the nAChRs being examined here are expressed on, as this is not characterized or stated clearly. The discussion does mention different cell types in this brain area, but it was not clear which neuronal subtype(s) the authors may be characterizing here.

8. It is not stated in the introduction what hypotheses there were regarding potential differences in nAChR functional changes at the different cellular locations. Why were these three locations quantified?

9. Related to the above point, given that dendritic and somatic nAChR function is enhanced after nicotine, the discussion should focus on why enhancement occurred in all three compartments.

10. What specific nAChR subunits do the authors hypothesize are being altered? This is not described in the manuscript.
